# New antigens for the serological diagnosis of human visceral leishmaniasis identified by immunogenomic screening

**DOI:** 10.1371/journal.pone.0209599

**Published:** 2018-12-20

**Authors:** Ana Maria Ravena Severino Carvalho, Tiago Antônio de Oliveira Mendes, Eduardo Antonio Ferraz Coelho, Mariana Costa Duarte, Daniel Menezes-Souza

**Affiliations:** 1 Programa de Pós-Graduação em Ciências da Saúde: Infectologia e Medicina Tropical, Faculdade de Medicina, Universidade Federal de Minas Gerais, Belo Horizonte, Minas Gerais, Brazil; 2 Departamento de Bioquímica e Biologia Molecular, Universidade Federal de Viçosa, Viçosa, Minas Gerais, Brazil; 3 Departamento de Patologia Clínica, COLTEC, Universidade Federal de Minas Gerais, Belo Horizonte, Minas Gerais, Brazil; University of Ostrava, CZECH REPUBLIC

## Abstract

Visceral leishmaniasis (VL) still represents a serious public health problem in Brazil due to the inefficiency of the control measures currently employed, that included early diagnosis and treatment of human cases, vector control, euthanasia of infected dogs and, recently approved in Brazil, treatment with Milteforam drug. Effective clinical management depend largely on early and unequivocal diagnosis, however, cross-reactivity have also been described in serological tests, especially when it refers to individuals from areas where Chagas’ disease is also present. Thus, to discover new antigens to improve the current serological tests for VL diagnosis is urgently needed. Here, we performed an immunogenomic screen strategy to identify conserved linear B-cell epitopes in the predicted *L*. *infantum* proteome using the following criteria: *i*) proteins expressed in the stages found in the vertebrate host, amastigote stage, and secreted/excreted, to guarantee greater exposure to the immune system; *ii*) divergent from proteins present in other infectious disease pathogens with incidence in endemic areas for VL, as *T*. *cruzi*; *iii*) highly antigenic to humans with different genetic backgrounds, independently of the clinical stage of the disease; *iv*) stable and adaptable to quality-control tests to guarantee reproducibility; v) using statistical analysis to determine a suitable sample size to evaluate accuracy of diagnostic tests established by receiver operating characteristic strategy. We selected six predicted linear B-cell epitopes from three proteins of *L*. *infantum* parasite. The results demonstrated that a mixture of peptides (Mix IV: peptides 3+6) were able to identify VL cases and simultaneously able to discriminate infections caused by *T*. *cruzi* parasite with high accuracy (100.00%) and perfect agreement (Kappa index = 1.000) with direct methods performed by laboratories in Brazil. The results also demonstrated that peptide-6, Mix III (peptides 2+6) and I (peptides 2+3+6) are potential antigens able to used in VL diagnosis, represented by high accuracy (Ac = 99.52%, 99.52% and 98.56%, respectively). This study represents an interesting strategy for discovery new antigens applied to serologic diagnosis which will contribute to the improvement of the diagnosis of VL and, consequently, may help in the prevention, control and treatment of the disease in endemic areas of Brazil.

## Introduction

Leishmaniasis are a complex group of diseases caused by protozoa parasites of the genus *Leishmania* and can lead, depending on the species, to two distinct diseases, namely visceral (VL) and tegumentary leishmaniasis (TL)[1–5]. VL is caused mainly by the parasites *L*. *donovani* and *L*. *infantum*, and it is estimated by the WHO that 200,000–400,000 new cases occur annually worldwide. *L*. *donovani* and *L*. *infantum* are the agents responsible for Old World VL, whereas *L*. *infantum* is responsible for New World VL [6]. VL is usually a systemic disease that affects internal organs, particularly the spleen, liver, and bone marrow [6, 7]. It is also considered a zoonosis, since it also affects dogs, with chronic evolution, systemic involvement and, if not treated, can lead to death in up to 90% of cases [3–5, 8]. Infected individuals that can not control the expansion of the parasites, develop the active form of the disease and may take severe and lethal forms, especially when associated with malnutrition and concomitant infections [7]. However, a significant proportion of residents in such areas can control parasite multiplication and remain asymptomatic in relation to VL [9]. Thus, it has been described that only 20% of patients infected by *L*. *infantum* develop symptomatic VL, whereas the vast majority of patients remain asymptomatic, a fact that difficult the identification of cases during clinical investigations and epidemiological analysis [10].

Regarding the laboratory diagnosis, VL can be diagnosed by tests that allow the direct or indirect detection of the infection by the parasite *L*. *infantum*. The parasitological diagnosis is highly specific, since it detects the presence of the parasite in aspirates of the spleen, lymph nodes or bone marrow of the patients through the visualization of amastigote forms in microscopy slides, detection of the DNA of the parasite by PCR, inoculum of the biopsies in culture media or in laboratory animals [11–13]. However, despite the high specificity, these methods has limitations in its sensitivity because the distribution of the parasite is not homogeneous in the tissues, which is higher in the spleen (94.7%) than in the bone marrow (64.3%) or in the lymph nodes (68.9%) [14]. Such factors also occur because of the low parasitic load present in these tissue samples depending on the stage of disease and the patient's immune status, which may lead to false negative results [15]. In addition, the collection of materials to perform these parasitological examinations is carried out by procedures considered to be invasive, depending on the technical expertise of the professional and also on the quality of the prepared slides, which complicate their use in routine medical practice [11–13].

In relation to indirect methods, serological diagnostic tests, for example, using enzyme-linked immunosorbent assay (ELISA), indirect immunofluorescence (IFI), Western blot, direct agglutination test (DAT) or immunochromatographic methods, based on the use of parasite extracts or recombinant, have been used for the serological diagnosis of VL and several diseases, because they are considered easy to perform, low cost and do not require laboratory infrastructure and high cost equipment [16]. In addition, there is an advantage in the improvement of such diagnostic condition, since the collection of samples is based on venipuncture to remove a small blood aliquot, being considered less invasive and with less risk to the patients in relation to biopsies for parasitological exams. However, several serological tests use preparations containing crude antigen from *Leishmania* parasite, that includes soluble antigens, and among the various antigens tested, variable sensitivity has been observed due to antigenic differences among parasite isolates and low specificity due to cross reactivity with other diseases that co-occur in endemic areas for VL, as Chagas' disease (CD)[17, 18].

Aiming to improve the sensitivity and specificity of the tests of VL, studies has used serological methods to detected antibodies with several antigens, including recombinant proteins, synthetic peptides and multiepitope proteins [19–24]. Among the most well-known antigens, recombinant protein K39 (rK39), which is currently used for the serodiagnosis of VL and CVL in several countries around the world, for example, is employing in the latex alglutination test methodologies (KAtex—Kalon Biological Limited, USA), direct agglutination test (DAT) or immunochromatographic (rK39 dipstick test—In Bios, USA) [25]. These methods showed sensitivity ranging from 87–98% and specificity of 89–98%. However, its validation for the detection of cases of *L*. *infantum* infection has varied according to the production of the recombinant protein and the clinical and serological variability of the patients, besides the genetic variability observed between different strains of the same species, especially when evaluated strains isolated from distant locations [25, 26]. These data indicate the urgent need to discover new antigens to improve the current serological tests for VL diagnosis and also canine visceral leishmaniasis (CVL).

Bioinformatic and immunoproteomic approaches has been used to discovery more sensitive and specific antigens [19, 20]. The strategy is based on *in silico* analysis of protein sequences to predict B-cell epitopes. In this sense, several antigens, as peptides, recombinant proteins and chimeric proteins were obtained to be used in VL and CVL diagnosis [21, 23]. Moreover, identification of reactive peptides on the surface of the phages and integration with immunoinformatics allows their use and/or the subsequent synthetic construction with a directed application for diagnosis [27, 28]. Thus, using the knowledge acquired in immunoinformatics for the discovery of targets for the diagnosis of infectious diseases [27, 29–31], and also for the limitations known for the serodiagnosis of VL, the objective to the present study was to identify and validate new antigens of *L*. *infantum* for the development of a biotechnological product applied to the serological diagnosis of this disease. For this, we selected six predicted linear B-cell epitopes from three proteins of *L*. *infantum* parasite, expressed in amastigote stage and higly divergent of the *T*. *cruzi* proteins. We also performed a comparative analyses with results obtained from an ELISA employing soluble *L*. *infantum* antigen (SLiA). The goal of this work to was: i) selected proteins expressed in the stages found in the vertebrate host, amastigote stage, and secreted/excreted, to guarantee greater exposure to the immune system; ii) that are divergent from proteins present in other infectious disease pathogens with incidence in endemic areas for VL, as *T*. *cruzi*; iii) highly antigenic to humans with different genetic backgrounds, independently of the clinical stage of the disease; iv) stable and adaptable to quality-control tests to guarantee reproducibility; v) and to use statistical analysis to determine a suitable sample size to evaluate accuracy of diagnostic tests established by receiver operating characteristic strategy.

## Materials and methods

### Ethics statement and human samples

The use of human samples was approved (protocol CAAE–60802116.1.0000.5138) by the Ethics Committee of the Federal University of Minas Gerais (UFMG), Belo Horizonte, Brazil. All patients received an individual copy of the study policy, which was reviewed by an independent person, and all participants gave their consent form in Portuguese, before blood collection. Procedures were performed with in accordance with the ethical standards and with the 1964 Helsinki declaration.

Sample size determination for accuracy of diagnostic tests was established using receiver operating characteristic (ROC curve) and PASS software (version 15, NCSS Statistical Software)[sample allocation ratio (R): R = Groups negative for VL/Groups positive for VL = 2/2 = 1]. A minimum sample of 67 from the positive group (VL) and 134 from the negative group (CT+CD) achieve 80% power to detect a difference of 0.05 between the area under the ROC curve (AUC) under the null hypothesis of 0.80 and an AUC under the alternative hypothesis of 0.85 using a two-sided z-test at a significance level of 0.05. The data are discrete (rating scale) responses. The AUC was computed between false positive rates of 0.00 and 1.00. The ratio of the standard deviation of the responses in the negative group to the standard deviation of the responses in the positive group was 1.00 [32, 33].

All sera were collected by venipuncture of medial vein in tubes without anticoagulant, and were kept at 37°C by 15 min, when they were centrifuged at 4000×*g* for 15 min, and the serum were separated and kept at −80°C until use. All samples are stored in a sera bank from Post-graduate Program in Infectious Diseases and Tropical Medicine, School of Medicine, UFMG. Samples from patients with visceral leishmaniasis (VL, *n* = 70) were confirmed by clinical evaluation associated by PCR technique targeting *L*. *infantum* kDNA in aspirates from spleen and/or bone marrow of the patients. The control group (CT, *n* = 70) consisted of healthy individuals without clinical signs, suspicious or positive laboratory tests for VL. Samples from patients with Chagas’ disease (CD, n = 68) were confirmed by clinical evaluation associated with hemoculture in combination with specific ELISA or indirect hemagglutination assay (IHA) assays [34].

### *In silico* prediction of linear B-cell epitopes

Initially, the predicted proteins based on the genome sequence of *L*. *infantum* were retrieved from database TritrypDB (8.1 version) [35]. Proteins containing at least two peptides identified previously in the intracellular mammalian stage amastigote by mass spectrometry were also retrieved from TritrypDB [35]. Epitopes (B-cells) were predicted using the BepiPred program (version 1.0) with cut-off set to 1.3 [36]. All peptide were identified using a conservative approach to minimize the selection of false-positive epitopes. Intrinsically unstructured/disordered regions (IURs) were considered as those with at least nine continuous amino acids with an individual score above 0.5 predicted by IUPred program [37]. Potential secreted/excreted proteins were predicted using the SignalP program version 4.1 [38] with organism group set to Eukaryotes, default parameter for D-cut-off values and method parameter with input sequences including TM regions.

In order to evaluate the specificity potential of peptides for diagnostic, the peptide sequences from *L*. *infantum* strain JPCM5 [39] were compared with encoded proteins based on genome sequences from *T*. *cruzi* (strains CL Brenner and DM28c) [40], *Leishmania* species (*L*. *donovani*, strain BPK282A1; *L*. *major*, strain Friedlin) [35] and *H*. *sapiens* assembly version GRCh37 from RefSeq database [41] using the BLASTp algorithm [42] with parameters optimized for short sequences analysis [43].

### Peptide synthesis

The selected peptides were chemical synthetized by GenScript company (New Jersey, United States) and. purified by high-performance liquid chromatography (HPLC). The molecular weights (MW) identified by mass spectrometry were consistent with the predicted theoretical MW. The lyophilized peptides were ressuspended in ultrapure water (Sigma- Aldrich) and stored at -20ºC until use in ELISA assays.

### Soluble *Leishmania infantum* antigen (SLiA)

*L*. *infantum* promastigotes (MHOM/BR/1974/PP75) were grown to stationary phase at 24ºC in Schneider’s insect medium (Sigma-Aldrich) supplemented with 10% inactivated fetal bovine serum (100 U/mL penicillin plus 100 μg/mL streptomycin and pH adjusted to 7.2). Previously, we performed PCR-restriction fragment length polymorphism (PCR-RFLP) to confirmed *L*. *infantum* specie [44]. A total of 1 x 10^10^ parasites were washed three times with cold phosphate buffered saline (PBS, pH adjusted to 7.2), followed by three cycles of freezing (liquid nitrogen) and thawing (42ºC). After ultrasonication with 10 alternating cycles of 30 s at 35 MHz, the lysate was centrifuged at 6,000x*g* at 4ºC for 15 min. The supernatant containing SLiA was collected and the protein concentration estimated using the Pierce BCA Protein Assay (Thermo Scientific).

### ELISA assays employing peptides based on linear B-cell epitopes

The peptides were coated onto flat-bottom plates (Costar, USA) overnight at 37°C at a concentration of 10 μg/well. All serum samples were tested by ELISA using each one of the peptides (1, 2, 3, 4, 5 and 6). In this study, the peptides 2, 3 and 6 showed high accuracy values (results section). Thus, we evaluated a mixture containing these peptides in the same proportion (Mix I: 2+3+6; 3.34 μg for each one/well). A mixture containing all peptides in the same proportion (Mix II: 1+2+3+4+5+6; 1.66 μg for each one/well) was evaluated. The ELISA assay was carried out as described previously [45]. To eliminates the need of combining three or more peptides at a time, we also evaluated the combination between peptides 2 and 6 (Mix III: 2+6; 5.0 μg for each one/well) and peptides 3 and 6 (Mix IV: 3+6; 5.0 μg for each one/well). The absorbance at 450 nm was read with an microplate reader (EMax, Molecular Devices, USA) and values were averaged and blank-corrected. The results of the ELISA using peptides as antigens, were compared with those using *L*. *infantum* SLiA-ELISA.

### Statistical analysis

The cut-off for peptides 1–6, Mix I, II, III, IV and SLiA were established for optimal sensitivity and specificity using the receiver-operator curve (ROC) curve. The cut-off was chosen based on the point that provided the maximum of the sum of the sensitivity and specificity [46]. The following parameters were calculated: sensitivity (Se), specificity (Sp), positive predictive value (PPV), negative predictive value (NPV), area under the curve (AUC), and accuracy (AC). Kolmogorov-Smirnoff normality method was used to to evaluate if the data have Gaussian distribution. The anti-peptides 1–6, Mix I, II, III, IV and SLiA reactivity between groups were compared by Kruskal-Wallis followed by Dunn’s post-test for multi-group comparisons (statistically significant at *p*<0.05). The degree of agreement between the ELISA assays with the parasitological test was determined by Kappa index (κ) values with 95% confidence intervals and interpreted according to the following Fleiss scale: 0.00–0.20, poor; 0.21–0.40, fair; 0.41–0.60, moderate; 0.61–0.80, good; 0.81–0.99, very good; and 1.00, perfect. The softwares GraphPad Prism^TM^ (version 5.0) and GraphPad QuickCals and GraphPad QuickCals (http://www.graphpad.com/quickcalcs/) were used in this step.

## Results

### Priorization of potential peptides for VL serodiagnosis

**Fig 1:**
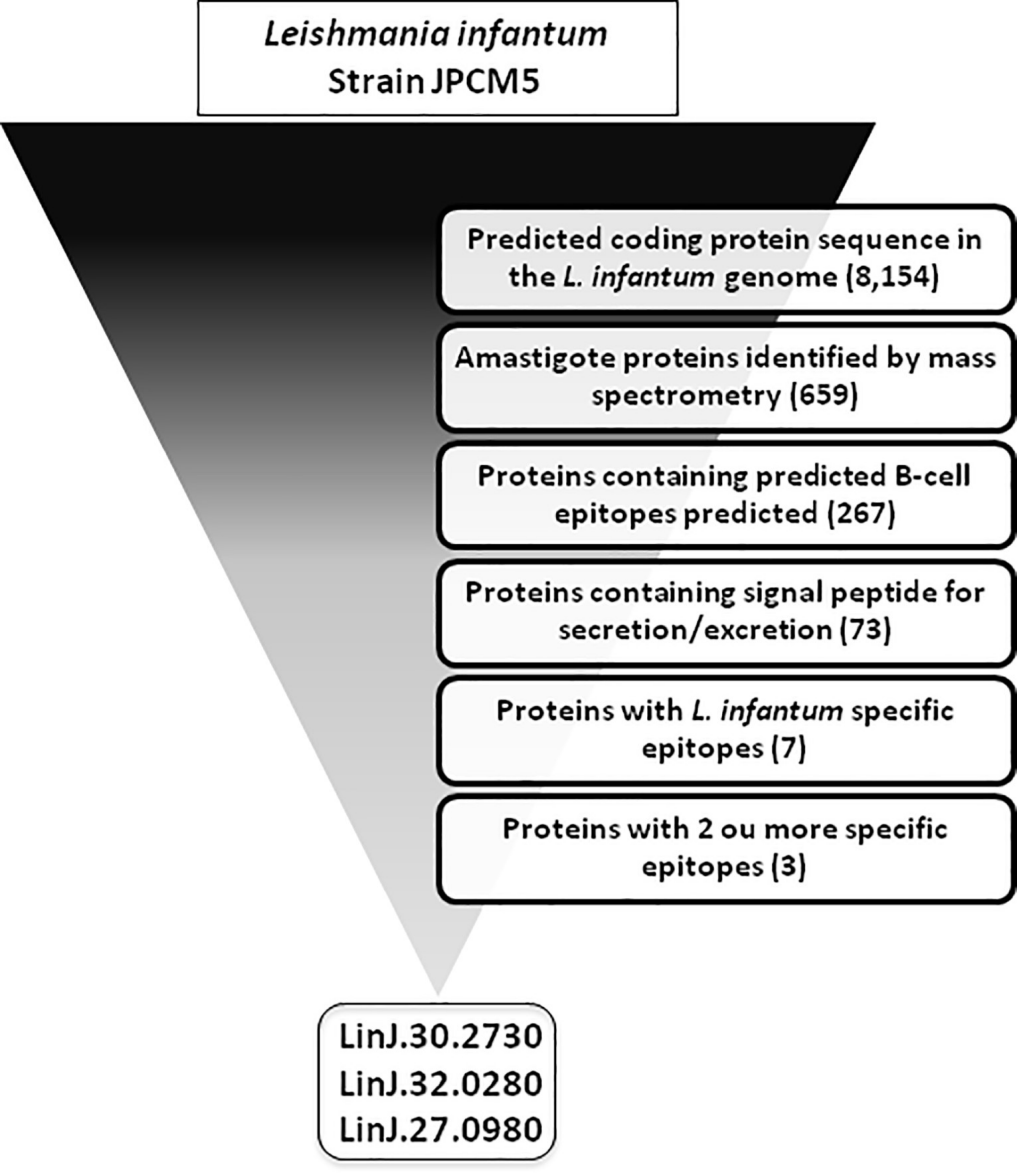
Flow chart with experimental designer of computational approach for identification of potential targets for VL serodiagnosis. *L*. *infantum* genome (strain JPCM5) displayed 8,154 predicted coding protein sequences. Potential targets were selected by following critera: protein evidence by mass spectrometry in stages found in the vertebrate host (amastigote); presence of predicted B-cell epitopes; potential excreted/secreted proteins; high specificity for *L*. *infantum* with reduced similarity to *T*. *cruzi* and human proteins; 2 or more specific epitopes.

**Fig 2:**
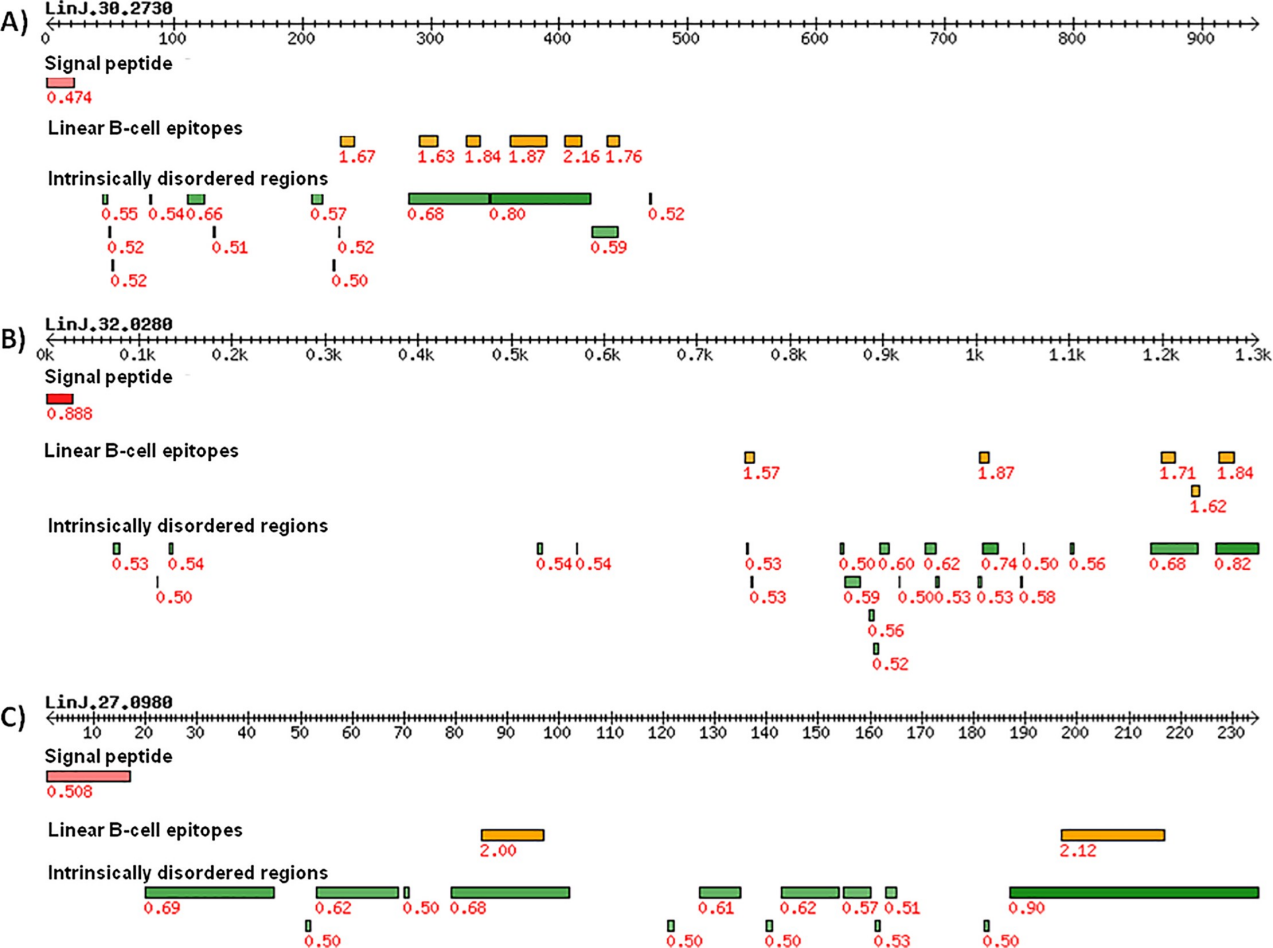
Top 3 secreted/excreted proteins from *L*. *infantum* amastigotes with predicted linear B-cell epitopes. The double arrow lines represent the primary structures of proteins with coordinates of amino acids as ticks. Red bars represent signal peptide for membrane export of proteins predicted by SignalP program. Yellow bars represent predicted linear B-cell epitopes with at least 9 amino acids predicted by Bepipred program. Green bars represent unstructured regions of proteins with epitopes available to interaction with antibodies predicted by IUPRED program. Red numbers represent prediction score for each program used. **A)** Gene LinJ.30.2730. **B)** Gene LinJ.32.0280. **C)** Gene LinJ.27.0980.

In order to identify potential antigenic and specific linear B-cell epitopes of *L*. *infantum*, a genome mining approach was used (Fig 1). A total of 659 proteins from the *L*. *infantum* genome were initially selected due to evidence of presence in amastigotes identified by mass spectrometry. A total of 267 proteins showed predicted linear B-cell epitopes and 73 out of them were predicted to be secreted/excreted by the parasite. The surface proteins containing epitopes are potentially more able for interacting more with B-cells than intracellular proteins and to induce antibodies against them. A total of 7 proteins showed low similarity with *T*. *cruzi* and human proteins and 3 with 2 ou more B-cell epitopes. The top 3 proteins were represented by genes annotated as: LinJ.30.2730 (stabilization of polarity axis, putative); LinJ.32.0280 (hypothetical protein, conserved); LinJ.27.0980 (guide RNA-binding protein of 21 kDa)(Fig 2).

### Epitope prediction combined with sequence divergence analysis suggest peptides 1–6 as potential diagnostic targets for VL

Two peptides predicted as linear B-cell epitope from each protein and specific for *L*. *infantum* were selected (Figs 1 and 2 and Table 1). The peptides chosen were: SGAPRANNSGDASA (peptide-1: Bepipred score = 1.63) and GLSGEGSPASPEPRLAGGGGGADTQSTT (peptide-2: Bepipred score = 1.87) from LinJ.30.2730; DGKPKENQKTARES (peptide-3: Bepipred score = 1.71) and VADSGSASSEDGGSAKP (peptide-4: Bepipred score = 1.84) from LinJ.32.0280; PRKADPNDTTPQ (peptide-5: Bepipred score = 2.00) and GDSPPSDSPQNNQDRNRNQN (peptide-6: Bepipred score = 2.12) from LinJ.27.0980. All peptides co-occur in intrinsically unstructured regions (IUPRED score: 0.68, 0.80, 0.68, 0.82, 0.68 and 0.90, respectively; Fig 2), which suggests that these proteins regions has an unfolded structure and is therefore potentially accessible for antibody binding.

**Table 1:** Peptides sequence, identity and similarity of the six B-cell linear epitopes predicted in the proteins of *L*. *infantum* and its orthologs in *Homo sapiens*, *T*. *cruzi* (strains CL Brenner and DM28c), *L*. *donovani* (strain BPK282A) and *L*. *major* (strain Friedlin).

Peptide*	Protein ID	Coordinate		Bepipred score	*H*. *sapiens*		*T*. *cruzi* CL Brenner		*T*. *cruzi* DM28c		*L*. *donovani* BPK282A1		*L*. *major* Friedlin	
		Inicial	Final		Id (%)	Si (%)	Id (%)	Si (%)	Id (%)	Si (%)	Id (%)	Si (%)	Id (%)	Si (%)
1	LinJ.30.2730	290	303	1.63	64.3	64.3	52.0	64.3	53.0	64.3	100.0	100.0	64.2	64.2
2	LinJ.30.2730	361	388	1.87	42.8	53.6	57.0	64.3	62.0	62.0	100.0	100.0	96.0	96.0
3	LinJ.32.0280	1198	1211	1.71	50.0	50.0	67.0	67.0	50.0	57.1	100.0	100.0	92.8	92.8
4	LinJ.32.0280	1259	1275	1.84	58.8	58.8	58.8	64.7	58.8	58.8	100.0	100.0	64.7	64.7
5	LinJ.27.0980	84	95	2.00	58.3	66.6	62.0	66.6	58.3	66.6	100.0	100.0	100.0	100.0
6	LinJ.27.0980	196	215	2.12	50.0	50.0	52.9	52.9	50.0	60.0	100.0	100.0	95.0	95.0

*Epitope sequence: Peptide-1, SGAPRANNSGDASA; Peptide-2, GLSGEGSPASPEPRLAGGGGGADTQSTT; Peptide-3, DGKPKENQKTARES; Peptide-4, VADSGSASSEDGGSAKP; Peptide-5, PRKADPNDTTPQ; Peptide-6, GDSPPSDSPQNNQDRNRNQN.

Abbreviations: Id (%), Identity (%); Si (%), Similarity (%).

Divergence with proteins encoded by their hosts and by parasites that co-occur in endemic areas for VL, in particular in the B-cell epitopes, may be sufficient to induce the production of *L*. *infantum*-specific antibodies [47]. In this context, we compared the sequences of predicted B-cell epitopes of peptide 1–6 with orthologous sequences from *T*. *cruzi* and *H*. *sapiens* (Table 1). For this, the specificity of peptides were confirmed by reduced identity and similarity with *T*. *cruzi* (strain CL Brenner: identity: 52.0–67.0%; similarity: 52.9–67.0%; strain DM28c: identity: 50.0–62.0%; similarity: 57.1–66.0%) and human proteins (identity: 42.8–64.3%; similarity: 50.0–66.6%). Interestingly, all peptides presented 100.0% of identity and similarity with peptides presented in proteins from *L*. *donovani* (strain BPK282A1), another etiological agent of VL. In relation to *L*. *major* (strain Friedlin), a TL specie, we observed moderate identity and similarity for peptides 1 and 4 (identity: 64.2–64.7%; similarity: 64.2–64.7%), while for 2, 3, 5 and 6 peptides (identity: 92.8–100.0%; similarity: 92.8–100.0%), the data showed high conservation of the sequences.

### Peptides based on linear B-cell epitopes from *L*. *infantum* proteins showed high performance in the diagnosis of chronic VL

**Fig 3:**
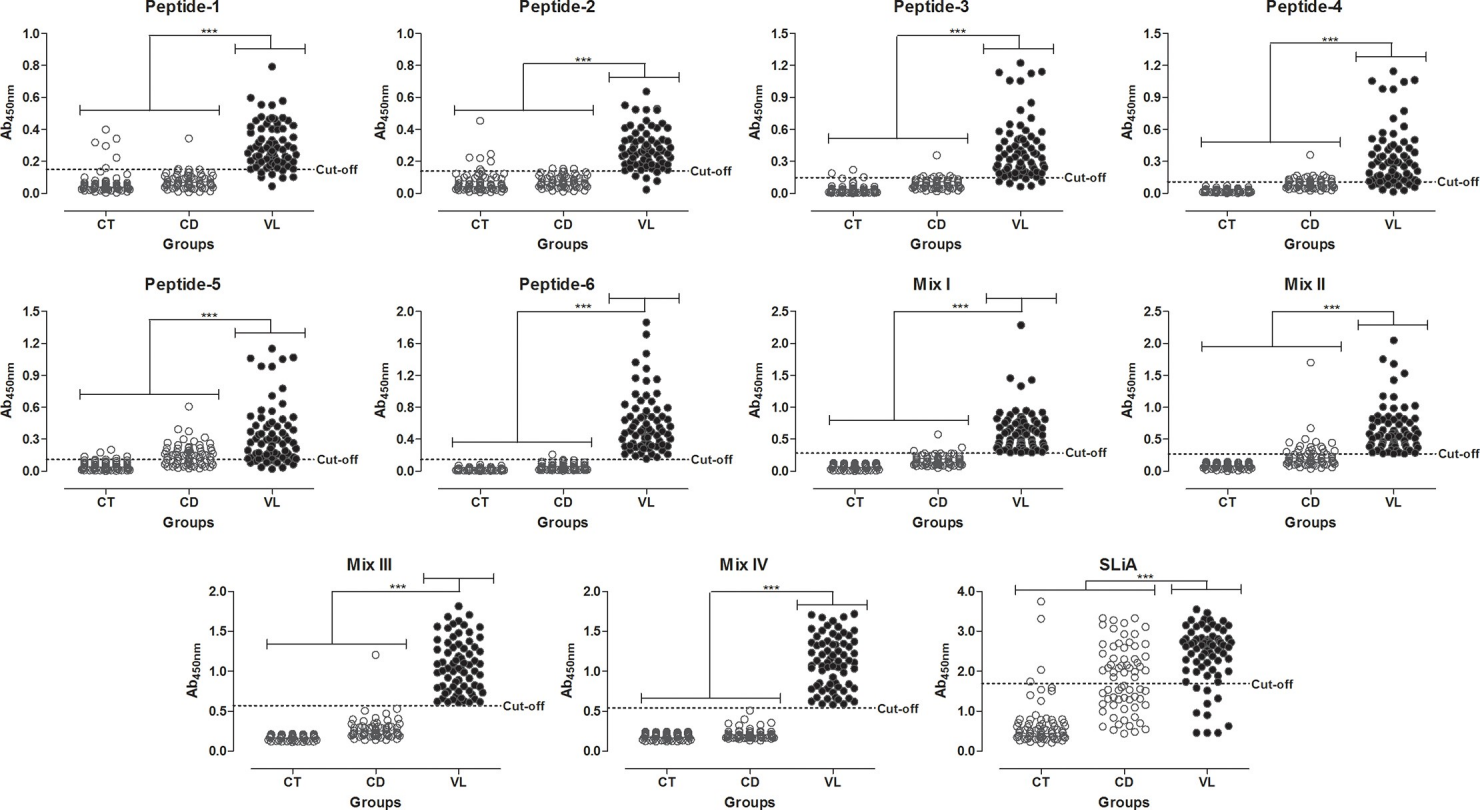
Comparison of reactivity from ELISA against Peptides 1–6, Mix I, II, III, IV and SLiA. ELISA was performed in different groups of individuals. Groups negative for VL (white circle): control group (CT, *n* = 70), Chagas’ disease (CD, *n* = 68). Groups positive for VL (black circle): visceral leishmaniasis (VL, n = 70). The anti-peptides 1–6, Mix I, II, III, IV and SLiA reactivity between groups were compared by Kruskal-Wallis followed by Dunn’s post-test for multi-group comparisons. The differences were considered statistically significant at ***p<0.001, highlighted by connecting lines. Cut-offs were determined by ROC curves.

**Fig 4:**
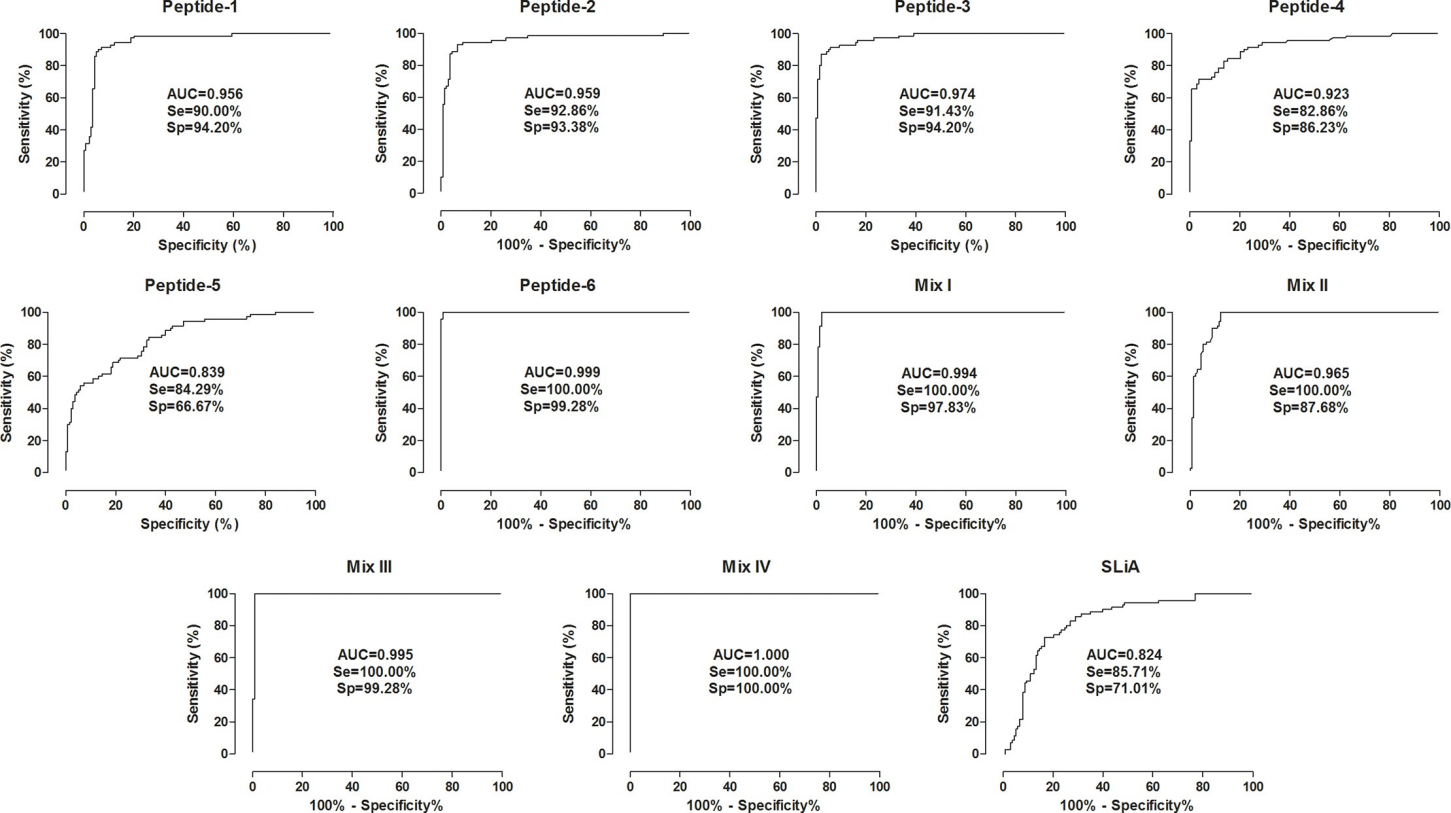
Comparison of ROC curves obtained from Peptides 1–6, Mix I, II, III, IV and SLiA. ROC-curve analysis was applied to define the appropriated cut-off to discriminate OD values from VL negative groups represented by healthy and *T*. *cruzi* infected patients (CT, *n* = 70; CD, *n* = 68) and VL positive groups (VL, *n* = 70). Additional performance parameters were also calculated and provided in the figure, including the area under the curve (AUC), the sensitivity (Se) and the specificity (Sp).

The performance of each antigen is summarized in Figs 3 and 4 and Table 2. Peptide-6, Mix I, II, III and IV showed value of 100.00% (CI 95%: 94.87–100.00%). Following, in descending order of the sensitivity values, we find the peptides 2, 3, 1, 5 and 4 (range: 82.86–92.86%). SLiA ELISA exhibited the sensitivity value of 85.7%. The maximum PPV was achieved by Mix IV (100.00%), followed by peptide-6 and Mix III (98.59%), Mix I (95.89%), peptide-3 (88.89%), peptide-1 (88.73%), peptide-2 (87.84%), Mix II (80.46%), peptide-4 (75.32%), SLiA (60.00%) and peptide-5 (56.19%).

**Table 2:** Measure of diagnostic performance for Peptides 1, 2, 3, 4, 5, 6, Mix I, II, III, IV and SLiA.

Parameters^*a*^	Diagnostic Test*										
	Peptide-1	Peptide-2	Peptide-3	Peptide-4	Peptide-5	Peptide-6	Mix I	Mix II	Mix III	Mix IV	SLiA
**TP**	63	65	64	58	59	70	70	70	70	70	60
**TN**	130	129	130	119	92	137	135	121	137	138	98
**FP**	8	9	8	19	46	1	3	17	1	0	40
**FN**	7	5	6	12	11	0	0	0	0	0	10
**Se (%)**	90.00	92.86	91.43	82.86	84.29	100.00	100.00	100.00	100.00	100.00	85.71
**Se-CI 95%**	80.48–95.88	84.11–97.64	82.27–96.79	71.97–90.82	73.62–91.89	94.87–100.00	94.87–100.00	94.87–100.00	94.87–100.00	94.87–100.00	75.29–92.93
**Sp (%)**	94.20	93.48	94.20	86.23	66.67	99.28	97.83	87.68	99.28	100.00	71.01
**Sp-CI 95%**	88.90–97.46	87.81–96.93	88.90–97.46	79.34–91.50	58.14–74.46	96.03–99.98	93.78–99.55	81.01–92.66	96.03–99.98	97.36–100.00	62.69–78.42
**PPV (%)**	88.73	87.84	88.89	75.32	56.19	98.59	95.89	80.46	98.59	100.00	60.00
**PNV (%)**	94.89	96.27	95.59	90.84	89.32	100.00	100.00	100.00	100.00	100.00	90.74
**AC (%)**	92.79	93.27	93.27	85.10	72.60	99.52	98.56	91.83	99.52	100.00	75.96
**AUC**	0.956	0.959	0.974	0.923	0.839	0.999	0.994	0.965	0.995	1.000	0.824
**AUC-CI 95%**	0.928–0.984	0.927–0.990	0.954–0.993	0.883–0.963	0.784–0.896	0.998–1.000	0.986–1.002	0.943–0.988	0.985–1.005	1.000–1.000	0.766–0.883
**κ**	0.839	0.851	0.850	0.674	0.454	0.989	0.968	0.827	0.989	1.000	0.513
**κ-CI 95%**	0.761–0.917	0.776–0.926	0.775–0.926	0.569–0.779	0.340–0.568	0.968–1.000	0.932–1.000	0.750–0.905	0.968–1.000	1.000–1.000	0.401–0.625
**Agreement**^***b***^	Very Good	Very Good	Very Good	Good	Moderate	Very Good	Very Good	Very Good	Very Good	Perfect	Moderate

^*a*^Parameters was calculated using all samples presented in this work: negative (CT+CD, n = 138) and positive for visceral leishmaniasis (VL, n = 70).

^*b*^Agreement was calculated using parasitological assays as gold standard test.

*Cut-off obtained by ROC curve.

Abbreviations: Mix I, peptides 2+3+6; Mix II, peptides 1–6; Mix III, peptides 2+6; Mix IV, peptides 3+6; TP, true positive; TN, true negative; FP, false positive; FN, false negative; Se, sensitivity; Sp, specificity; CI, confidence interval; PPV, predictive positive value; PNV, predictive negative value; AC, accuracy; AUC, area under curve; κ, kappa index.

To further evaluate the diagnostic specificity of the peptides, sera from control individuals or patients with Chagas' disease were tested. Mix IV showed the higher specificity value (100.00%, CI 95%: 97.36–100.00), followed by Peptide-6 and Mix III (99.28%, CI 95%: 96.03–99.98), Mix I (97.83), peptides 1 and 3 (94.20%), peptide-2 (93.48%), Mix II (87.68), peptide-4 (86.23%), SLiA (71.01%) and peptide-5 (66.67%). Maximun NPV values were observed for peptide-6, Mix I, II, III and IV (100.00%), followed by peptide-2 (96.27%), peptide-3 (95.59%), peptide-1 (94.89%), peptide-4 (90.84%), SLiA (90.74%) and peptide-5 (89.32%).

Mix IV showed the best diagnostic value for VL represented by greater accuracy value, followed by Peptide-6 and Mix III (99.52%), Mix I (98.56%), peptide-2 and 3 (93.27%), peptide-1 (92.79%), Mix II (91.83%), peptide-4 (85.10%), SLiA (75.96%) and peptide-5 (72.60%). Analysis of area under curve (AUC) using ROC curves showed and confirmed the better performance of Mix IV (AUC = 1.000, CI 95% 1.000–1.000) as compared to the other antigens, as described in order of the best values: Peptide-6 = 0.999; Mix I = 0.994; Peptide-3 = 0.974; Mix II = 0.965; Peptide-2 = 0.959; Peptide-1 = 0.956; Peptide-4 = 0.923; Peptide-5 = 0.839; SLiA = 0.824.

In addition, we showed that the VL patients showed high values for specific antibodies against all peptides, Mix I, II, III, IV and SLiA when compared to all non-infected groups (CT and CD; *p*<0.001).

### Mix IV, Peptide-6 and Mix I showed high agreement with the parasitological test evaluated by Kappa index

The agreement (Kappa index, κ) between the peptides 1–6, Mix I, II, III, IV and SLiA with the parasitological assays (direct method) is shown in Table 2. Mix IV ELISA displayed the best agreement value of all tests with a perfect concordance in comparison to parasitological assays (κ = 1.000, CI 95%: 1.000–1.000). Peptide-6 and Mix I showed high agreement values (κ = 0.989, CI 95%: 0.968–1.000; κ = 0.968, CI 95%: 0.932–1.000; respectively). Very good agreement also was observed for peptides 1–3 and Mix II ELISA, however, with lower values than observed in peptide-6 and Mix I (κ = 0.839, CI 95%: 0.761–0.917; κ = 0.851, CI 95%: 0.776–0.926; κ = 0.850, CI 95%: 0.775–0.926; κ = 0.827, CI 95%: 0.750–0.905; respectively). A good agreement was observed for peptide-4 (κ = 0.674, CI 95%: 0.569–0.779) while peptide-5 showed a moderate (κ = 0.454, CI 95%: 0.340–0.568). When evaluating ELISA assay employing SLiA, we observed moderate agreement (κ = 0.513, CI 95%: 0.401–0.625).

## Discussion

VL still represents a serious public health problem in Brazil due to the inefficiency of the control measures currently employed, represented by the incidence of 3,453 cases in 21 states of Brazil (2014). In addition, in the same year, the incidence rate was 0.68 cases per 100,000 inhabitants and 87 cases of deaths associated with the disease, and in recent years, lethality has been increasing gradually, from 3.2% in 2000 to 6.6% in 2014 (Sinan/SVS/MS, http://portalsinan.saude.gov.br/dados-epidemiologicos-sinan). In this sense, considering the importance of VL as a serious public health problem, control measures to combat this disease involves: early diagnosis and treatment of human cases, spraying of insecticides with residual effect at home and next to residences, with the purpose of vector control, and euthanasia of infected dogs. Due to the limited antileishmanial drugs available for treatmenf of VL, effective clinical management depend largely on early and unequivocal diagnosis. However, regarding the diagnosis of human cases, problems of cross-reactivity have also been described in serological tests, especially when it refers to individuals from areas where Chagas’ disease is also present [27]. These data indicate the urgent need to discover new antigens to improve the current serological tests for VL diagnosis.

In order to solve the problems of sensitivity and/or specificity, as described above, studies has been carried out to identify new antigens candidates based on experimental approaches evaluating *in silico* prediction data by immunoinformatics and published genomes [27, 45]. This strategy allows the rational search for antigenic proteins, as well as to determine the regions of these targets that determine the antigenicity and specificity for the recognition of the targets by sera from patients [27, 48]. In this study, the proposal was to discover new potential antigens for use in serologic diagnosis of VL using the following criteria: proteins expressed in the stages found in the vertebrate host, amastigote stage, and secreted/excreted, to guarantee greater exposure to the immune system; divergent from proteins present in other infectious disease pathogens with incidence in endemic areas for VL, as *T*. *cruzi*; highly antigenic to humans with different genetic backgrounds, independently of the clinical stage of the disease; stable and adaptable to quality-control tests to guarantee reproducibility.

For this, we performed an immunogenomic screen strategy to identify conserved linear B-cell epitopes in the predicted proteome based on the genome sequence from *L*. *infantum*. Three proteins were selected, all expressed in amastigote stage identified by mass spectrometry with signal peptide for secretion/excretion and with two or more B-cell epitopes. Immunoproteomic and bioinformatic approaches, secreted antigens presented in the most common *Leishmania* species in Brazil, showed a potential targets for diagnostic for CVL diagnosis [49]. Choice of proteins expressed in specific stages of the parasites found in the vertebrate host is a determining factor in the selection of serological targets, due to greatest exposure to the host's immune system. The increase of the levels of the protein in these stages can suggest its involvement as parasite virulence factor [48]. Data revealed that the genes encoding the potential peptides are expressed in intracellular parasite stage amastigote, thus, could be exposed to the host cells and immune system and induce high production of antibodies. In fact, evaluating peptides ELISA assays, high sensitivity values were observed, that ranging from 82.86–100.00%, included peptide mixtures (Mix I, II, III and IV), and can be explained by high expression in intracellular stages, subcellular localization (secreted/excreted) and high antigenicity demonstrated by B-cell prediction. In according, previous studies have demonstrated that proteins expressed in stages found in vertebrate host are possibly associated with with the infection process and/or the intracellular survival of the parasite within the host, and may be attractive diagnostic targets [30, 45, 49]. Serological tests have also presented limitations regarding the absence of correlation between the antibody levels and the current stage of the disease [21, 50]. Other adictional factor is the divergence with host proteins, here evaluated by identity and similarity (identity: 42.8–64.3%; similarity: 50.0–66.6%) analysis between *L*. *infantum* and *H*. *sapiens* proteins, which avoid the possibility of the targets being similar to the host's self-antigens, guaranteeing a higher production of antibodies and, consequently, greater sensitivity [45].

Another determining factor in the selection of serological targets is the specificity. Part of the specificity problems of the target proteins could be solved by the identification of B-cell epitopes, with a high score, and analysis of the divergence of these regions with targets present in the host and in microorganisms that have cross-reactivity with VL [27, 47]. The use of synthetic peptides as antigens in diagnosis of VL may limit cross reactivity, as described to, B-cell epitopes mapped by immunoinformatic approaches [20, 24, 51]. Here, we selected divergent epitopes in relation to proteins enconded by *T*. *cruzi* parasite, confirmed by reduced identity (50.0–67.0%) and similarity (52.9–67.0%). In fact, the divergent as compared to *T*. *cruzi* proteins allowed to obtain excellent specificity values, mainly when reference to Mix IV (Sp = 100.00%), peptide-6 and Mix III (Sp = 99.28%), and Mix I (Sp = 97.83%).

A variety of antigens has been tested for the serodiagnosis of VL, such as recombinant proteins, synthetic peptides, phage clones, chimeric proteins and peptides/proteins mixtures [20–24, 51–53]. Mixture of antigens have already been used for the diagnosis of leishmaniasis, showing promising results. In our study, Mix IV, peptide-6, Mix III and Mix I were recognized by sera from VL patients and able to discriminate healthy individuals or infected by others trypanosomatids (accuracy = 100.00% and AUC = 1.000; accuracy = 99.52% and AUC = 0.999; accuracy = 99.52% and AUC = 0.999; accuracy = 98.56% and AUC = 0.994; respectively). Thus, Mix IV is the best peptide-6 and Mix I showed the excellent general performance in the diagnosis of VL as compared to all peptides. Thus, we showed that the mixture of peptides increased the values of accuracy when compared to the isolated, confirmed by the highest performance values for the Mix IV and III. Chimeric proteins, containing multipitopes from peptides and discovery by immunoinformatics and/or immunoprotemic, has been previously used as an efficient strategic to increase the accuracy of diagnostic tests [20, 24].

In Brazil, most of the conventional serological tests, as ELISA and indirect immunofluorescence assay (IFA), use a complex mixture of crude *Leishmania*-antigen preparations or the entire parasite itself, respectively. Those antigens show variable sensitivity due to antigenic differences among *Leishmania* isolates and lack specificity due to cross-reactivity with other diseases, mainly associated between *Leishmania spp*. and *T*. *cruzi* by the sharing of antigens by phylogenetic proximity [27, 45]. Sensitivity and specificity values for leishmaniasis detection in ELISA ranges depending on the antigen preparation for adsorption of the well plate [54, 55]. In according, with evaluated ELISA using soluble *L*. *infantum* antigen, SLiA, as antigen, very low performance values were observed. Thirty-six false-positive individuals were identified in CD group and four in controls individuals (CT).

Finally, this study identified different antigenic *L*. *infantum* peptides, based on linear B-cell epitopes, with potential for the development of the an immunoassay compound of isolated or multiantigens able to improve the sensitivity and specificity values for the VL serodiagnosis. The results demonstrated that synthetic Mix IV, peptide-6, Mix III and Iwere able to identify VL cases and simultaneously able to discriminate infections caused by *T*. *cruzi* parasite with high accuracy and agreement (Kappa index = 1.000; 0.989; 0.989 and 0.968, respectively) with direct methods performed by laboratories in Brazil. The perspective of the present study is to make the screening in asymptomatic individuals of endemic areas to make agreement with direct methods. This study represents an interesting strategy for discovery new antigens applied to serologic diagnosis which will contribute to the improvement of the diagnosis of VL and, consequently, may help in the prevention, control and treatment of the disease in endemic areas of Brazil.
